# Comprehensive analysis of genetic and clinical characteristics of 30 patients with X‐linked juvenile retinoschisis in China

**DOI:** 10.1111/aos.14642

**Published:** 2020-10-30

**Authors:** Feng‐Juan Gao, Jian‐Hong Dong, Dan‐Dan Wang, Fang Chen, Fang‐Yuan Hu, Qing Chang, Ping Xu, Wei Liu, Jian‐Kang Li, Ying Huang, Ji‐Hong Wu, Ge‐Zhi Xu

**Affiliations:** ^1^ Eye Institute Eye and ENT Hospital College of Medicine Fudan University Shanghai China; ^2^ Shanghai Key Laboratory of Visual Impairment and Restoration Science and Technology Commission of Shanghai Municipality Shanghai China; ^3^ Key Laboratory of Myopia (Fudan University) Chinese Academy of Medical Sciences National Health Commission Shanghai China; ^4^ Department of Ophthalmology Central Hospital of Xuhui District Shanghai China; ^5^ BGI‐Shenzhen Shenzhen China; ^6^ Laboratory of Genomics and Molecular Biomedicine Department of Biology University of Copenhagen Copenhagen Denmark; ^7^ Shenzhen Engineering Laboratory for Birth Defects Screening BGI‐Shenzhen Shenzhen China; ^8^ Department of Computer Science City University of Hong Kong Kowloon Hong Kong SAR China

**Keywords:** Chinese population, clinical diagnosis, molecular genetics, optical coherence tomography, *RS1*, X‐linked retinoschisis

## Abstract

**Purpose:**

To provides the clinical and genetic characteristics of a series of Chinese patients with X‐linked juvenile retinoschisis (XLRS) through multimodal imaging and next‐generation sequencing.

**Methods:**

Thirty patients (60 eyes) from 29 unrelated families of Chinese origin with XLRS were screened using multigene panel testing, and underwent a complete clinical evaluation. All variants identified in this study and reported in the Human Gene Mutation Database were analysed.

**Results:**

Twenty‐five distinct variants in the retinoschisin gene were identified, of which eight were novel, and one was *de novo*. Missense mutations were the most prevalent type, and mutation hot spot was localized in the discoidin domain. The mean Snellen best‐corrected visual acuity was 0.28 ± 0.17. Of all eyes presenting with schisis, 92.86% had lamellar schisis and 62.5% had peripheral schisis. Schisis changes mostly involved inner and outer nuclear layers. X‐linked juvenile retinoschisis (XLRS) patients had a high incidence of complications, and peripheral schisis was a risk factor for it. No obvious genotype–phenotype association was observed.

**Conclusion:**

This study provides comprehensive analyses of the genetic and clinical characteristics of XLRS in a cohort of Chinese patients. The fourth de novo mutation in RS1 was identified. And we show that XLRS has a wide spectrum of clinical characteristics; hence, molecular diagnosis is crucial for its diagnosis, differential diagnosis and genetic counselling. Peripheral schisis is a risk factor for the high incidence of complications, and no clear genotype–phenotype correlations were found.

## Introduction

X‐linked juvenile retinoschisis (XLRS; OMIM 312700) is a common early‐onset X‐linked recessive disease, with a prevalence ranging between 1 in 5000 and 1 in 25 000 (Molday et al. 2012). It is characterized by bilateral macular involvement with onset in the first decade of life, sometimes as early as 3 months of age (Renner et al. 2008). The schisis primarily occurs in the retinal nerve fibre layer, including the inner nuclear layer (INL), outer nuclear layer (ONL) and ganglion cell layer (GCL) (Gehrig et al. 1999a; Gehrig et al. 1999b; Li et al. 2007). X‐linked juvenile retinoschisis (XLRS) is categorized into four types: foveal schisis (FS) patients have FS only; foveo‐lamellar schisis (FLS) patients have FS and lamellar schisis (LS), but not peripheral schisis (PS); complex type disease (FLPS) includes patients with foveal, lamellar and PS; and foveo‐peripheral type patients (FPS) have foveal and PS, without LS (Prenner et al. 2006). The penetrance of XLRS is almost complete, but clinical expression is highly variable (Molday et al. 2012), resulting in an unpredictable disease progression and severity even within families. Visual acuity varies greatly among affected males, typically from 20/60 to 20/120, and remains relatively stable until the patient reaches their fifth decade (Pimenides et al. 2005). During the course of the disease, secondary complications including retinal detachment (RD), macular holes (MH), vitreous haemorrhage (VH) and neovascular glaucoma may occur, leading to a poor outcome (Ip et al. 1999; Pimenides et al. 2005). Swept‐domain optical coherence tomography (SD‐OCT) is the main diagnostic technique for this disease (Renner et al. 2008; Yu et al. 2010).

The retinoschisin gene (*RS1*; OMIM 300839), located on Xp22.13, is the only gene known to be associated with XLRS. It contains six exons and encodes a 224 amino acid protein. Retinoschisin protein is organized into four distinct regions, including an N‐terminal signal sequence characteristic of secreted proteins (exons 1 and 2; 23 amino acids), a retinoschisin domain (exon 3; 39 amino acids), a well‐conserved discoidin domain (exons 4–6; 157 amino acids) implicated in cell adhesion, and a C‐terminal segment (end of exon 6; five amino acids) (Gehrig et al. 1999a; Gehrig et al. 1999b). Retinoschisin protein is predominantly expressed and secreted from photoreceptor cells and most bipolar cells (Molday et al. 2001). It is an extracellular adhesion protein that plays a crucial role in maintaining the structural integrity of the retina (Molday et al. 2001).

A broad spectrum of clinical phenotypes has been reported for XLRS patients (Ip et al. 1999; Molday et al. 2012), making clinical diagnosis challenging, especially for young patients. Genetic testing is an effective method for providing timely, rapid and accurate diagnoses (Splinter et al. 2018), and multimodal imaging is an excellent technique for confirming phenotypes and helping clinicians understand the disease (Shinohara et al. 2018). The present study was aimed to report and analyse the genetic and clinical characteristics of XLRS in a cohort of Chinese patients, to advance our understanding of the disease. These data facilitate genetic counselling and the selection of patients who are eligible for gene augmentation.

## Materials and Methods

### Subjects and ethics statement

Participants were screened in the eye genetic disease clinic of the Eye and ENT Hospital of Fudan University (Shanghai, China) between January 2017 and March 2019. This study was approved by the Ethics Committee of the Eye and ENT Hospital of Fudan University, and adhered to the tenets of the Declaration of Helsinki. All patients or their guardians provided written informed consent.

### Genetic analysis and variant assessment

Next‐generation sequencing was performed in all patients and available family members. Genomic DNA was extracted from whole peripheral blood using the FlexiGene DNA Kit (Qiagen, Venlo, the Netherlands) according to the manufacturer's protocol. All participants underwent sequencing with a 762 gene panel (including genes involved in common inherited eye diseases; Table S1; BGI, Shenzhen, China) using BGISEQ‐2000. We aligned sequence reads to the reference human genome (UCSC hg 38) with the Burrows–Wheeler Aligner version 0.7.10 (Huang et al. 2017). The bioinformatics pipeline was applied as previously reported (Huang et al. 2017; Gao et al. 2019a; Gao et al. 2019b). Previously reported variants were determined using the Human Gene Mutation Database (HGMD, professional version 2019.1). Variants were classified according to the American College of Medical Genetics (Stenson et al. 2012). Sanger sequencing was performed to evaluate the presence of variants within other members in the family.

### Clinical assessment

Participants underwent comprehensive ophthalmic examinations after detailed family histories were obtained, including Snellen best‐corrected visual acuity (BCVA), slit‐lamp biomicroscopic ophthalmoscopy examination, intraocular pressure (Goldmann tonometry), dilated fundoscopy, colour fundus photography (Topcon TRC50LX; Topcon, Tokyo, Japan), ultra‐wide‐field scanning laser ophthalmoscopy (Optos 200Tx; Optos, Dunfermline, UK), SD‐OCT (Heidelberg Engineering, Heidelberg, Germany) and full‐field electroretinography (ffERG, according to the standards of the International Society for Clinical Electrophysiology of Vision available at www.iscev.org). Electroretinography (ERG) measurements were performed on 200 healthy subjects (age range, 3–50 years; median, 18 years) as control values.

## Results

### Cohort characteristics

Accurate genetic diagnosis was made after pedigree cosegregation and phenotypic confirmation. A total of 30 patients from 29 unrelated families of Chinese origin with a genetic diagnosis of XLRS were recruited. All patients were male, and both eyes were affected. The mean age was 15.73 ± 12.13 years (range, 4–48 years; median, 10 years; Table 1).

**Table 1:** Genotype and phenotype of the 30 XLRS patients.

Mutation type	Nucleotide and amino acid changes	ACMG category and references	Subjects (age/years)	BCVA (R/L)	XLRS type (R/L)	Schisis localization	Complications
Missense	c.577C>A p.Pro193Thr	LP Novel	F1(34)	0.15/0.1	FLPS/FLS	OU: INL/ONL/OPL/GCL	POAG (OU), VVs (R)
	c.304C>T p.Arg102Trp	P (Sauer et al. 1997)	F2(25)	0.1/0.02	FLPS/FLPS	OU: INL/ONL	OU: PACG, RPEAM, cataract L: RD. R: VVs
			F8(9)	0.25/0.4	FLPS/FLPS	OU: INL/GCL	OU: VAAs and exudations
	c.625C>T .Arg209Cys	P (1998)	F6(48)	0.2/0.3	MA/MA	–	OU: RPEAM, VAAs and exudations
	c.554C>T p.Thr185Met	LP Novel	F7(33)	0.1/0.06	FLS/FLS	OU: ONL/INL	Morning glory (OU)
	c.638G>A p.Arg213Gln	P (Hotta et al. 1998)	F9(10)	0.4/0.2	FLS/FLS	R: INL/GCL L: INL/ONL/GCL	IMH (L), POAG (OU)
	c.325G>A p.Gly109Arg	P Novel	F10(19)	0.5/0.4	FLS/FLS	OU: INL/OPL/GCL	N
	c.336G>T p.Trp112Cys	P (1998)	F11(40)	0.15/0.3	MA/FLS	R: – L: INL/ONL/GCL	IMH (L)
	c.214G>A p.Glu72Lys	P (1998)	F12(15)	0.3/0.25	FLPS/FLPS	R: INL/OPL/GCL L: INL/ONL/OPL/GCL	OU: VVs, RPEAM, VAAs and exudations
			F19(6)	0.12/0.15	FLPS/FLPS	OU: INL/ONL/GCL	VAAs (L)
	c.416 A>G p.Gln139Arg	LP Novel	F14(10)	0.4/0.4	FLPS/FLPS	R: INL/ONL/OPL L: INL/ONL	OU: VVs, VAAs and RPEAM
	c.590G>A p.Arg197His	P (1998)	F15(5)	0.25/0.4	FLS/FLPS	OU: INL/ONL/OPL/GCL	L: VAAs and RPEAM
	c.544C>T p.Arg182Cys	P (1998)	F16(4)	0.3/0.5	FLPS/FLPS	OU: INL/ONL/OPL	R: RPEAM
			F27(34)	0.08/0.3	MA/FLS	R: – L: INL/ONL	R: VAAs, RPEAM
	c.575C>T p.Pro192Leu	P (Riveiro‐Alvarez et al. 2007)	F17(22)	0.12/0.12	FLPS/FLPS	OU: INL/ONL/OPL/GCL	OU: VVs, VAAs, IRK and exudations
	c.626G>A p.Arg209His	P (Sauer et al. 1997)	F20(17)	0.4/0.4	FLPS/FLPS	OU: INL/ONL/OPL/GCL	OU: VVs, VAAs, RPEAM and exudations
	c.656G>A p.Cys219Tyr	LP Novel	F21(10)	0.7/0.4	FLPS/FLPS	OU: INL/ONL/OPL/GCL	OU: VVs and RPEAM. L: VAAs, IRK
	c.421C>T p.Arg141Cys	P (1998)	F22(8)	0.15/0.2	FLS/FLS	OU: INL/ONL/OPL/GCL	N
	c.626G>T p.Arg209Leu	P (Skorczyk & Krawczynski 2012)	F24(5)	0.3/0.4	FLPS/FLPS	OU: INL/ONL/OPL/GCL	OU: VVs, VAAs. L: RPEAM and exudations. R: VH
	c.312C>G p.Asn104Lys	P (Huopaniemi et al. 1999)	F28(12)	0.7/0.5	FLS/FLPS	R: INL/ONL/OPL/GCL L: INL/ONL/GCL	L: RPEAM, VVs
Splicing	c.78+5G>T	P (Chen et al. 2014)	F29(5)	0.4/0.4	FLPS/FLPS	OU: INL	OU: VVs, VAAs, RPEAM and exudations
	c.52+2T>A	P Novel	F18(4)	0.1/0.25	FLS/FLS	OU: INL/ONL	N
Frameshift	c.97delT p.Trp33GlyfsX93	P (Bowles et al. 2011)	F30(5)	0.25/0.3	FLS/FLS	OU: INL/ONL	N
			F26(7)	0.2/0.3	FLPS/FLPS	OU: INL/GCL	OU: VVs, VAAs, RPEAM. L: RPEAM
	c.573delG p.Ile194Serfs*43	P (Hewitt et al. 2005)	F3(24)	0.3/0.3	FS/FS	OU: INL/ONL/OPL/GCL	N
			F4(7)	0.5/0.5	FPS/FPS	OU: INL/ONL/OPL	strabismus (OU)
	c.14 delT p.Ile5LysfsX121	LP Novel	F5(27)	0.05/0.25	FLPS/FLS	OU: INL/ONL/OPL/GCL	R: VAAs, IRK and RPEAM
	c.577_579del p.Ter193del	LP Novel	F25(9)	0.05/0.15	FLPS/FLPS	OU: INL/ONL	OU: VVs, VAAs and IRK. L: Retinal white flecks. R; VAAs, cataract and RPEAM and exudations
Gross deletion	EX1‐3DEL	P (Chu et al. 2013)	F13(9)	0.8/0.05	FLPS/FLPS	R: INL/ONL/OPL/GCL L: INL/ONL/OPL	L: VH, RD, cataract OU: VVs, VAAs
	EX4‐6DEL	P (Bowles et al. 2011)	F23(9)	0.3/0.03	FLS/FLPS	R: INL/ONL/OPL/GCL L: INL/ONL	L: VVs and RPEAM

– = no schisis, BCVA = best‐corrected visual acuity, FLPS = foveo‐lamellar schisis, plus peripheral schisis, FLS = foveo‐lamellar schisis, FS = foveal schisis, GCL = ganglion cell layer, IMH = inner macular hole, INL = inner nuclear layer, IPL = inner plexiform layer, IRK = inner retinal break, L = left, LP = likely pathogenic, MA = macular atrophy, N = normal, ONL = outer nuclear layer, OPL = outer plexiform layer, OU = binocular, P = pathogenic, PACG = primary angle closure glaucoma, POAG = primary open closure glaucoma, R = right, RD = retinal detachment, RPEAM = retinal pigment epithelial alteration or mottling, VAAs = vascular abnormalities, VH = vitreous haemorrhage, VVs = vitreous veils, XLRS = X‐linked juvenile retinoschisis.

### Genetic findings

**Fig. 1:**
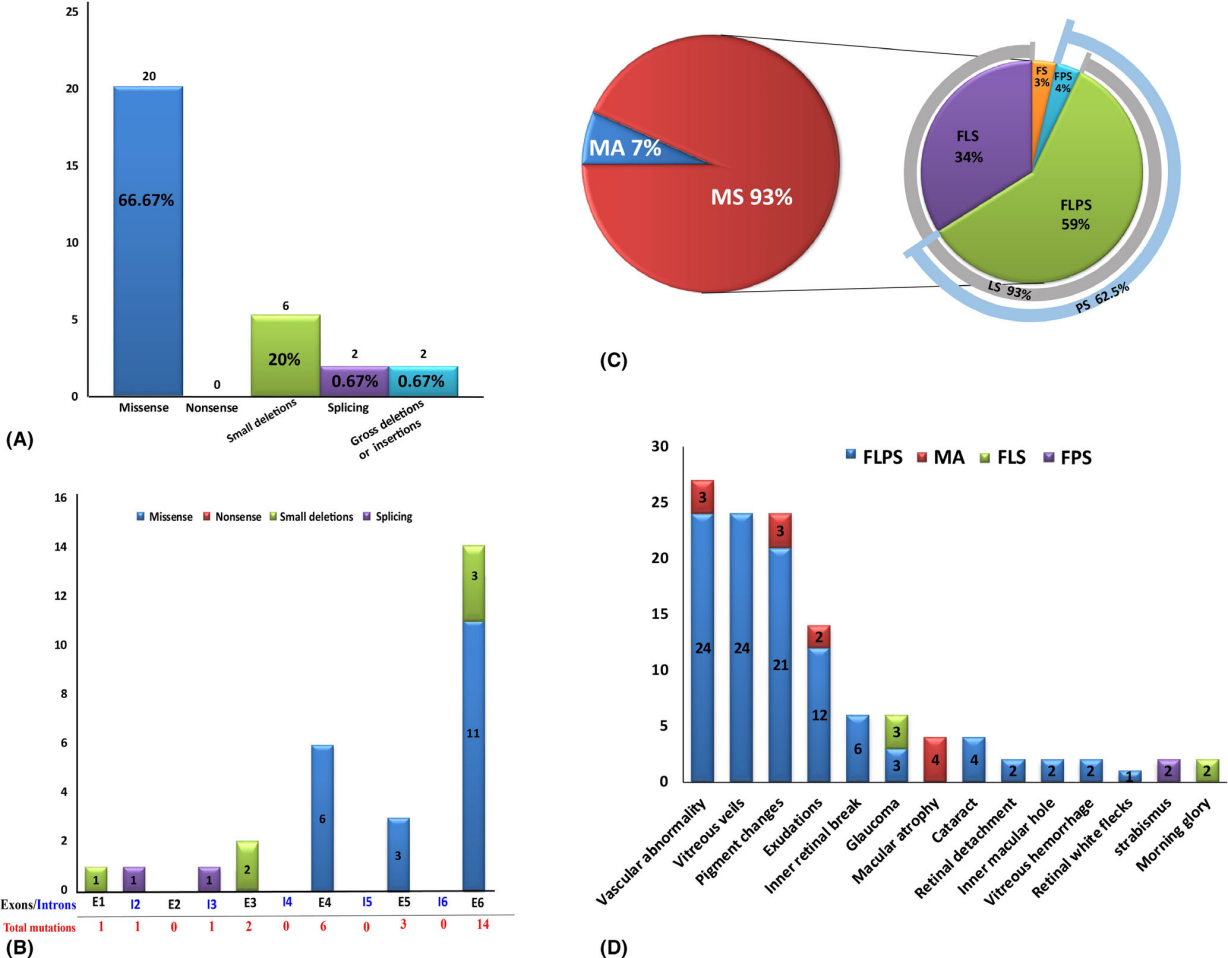
Overview of pathologic *RS1* mutations identified in this study and complications of all the patients. (A) Number of *RS1* mutations of different mutation types identified in this study. (B) Distribution of the *RS1* variants identified in this study. Exons are numbered in black font, and introns are numbered in blue font. Total mutations are shown in red font. (C) Pie chart showing the structural changes in XLRS patients. Macular schisis was seen in 93% of patients, with lamellar schisis (LS) accounting for 93% and peripheral schisis (PS) accounting for 62.5%. (D) Distribution of the different complications observed in different types of patients. FLPS = complex type patients who had foveal, lamellar and peripheral schisis., FLS = foveo‐lamellar patients who had foveal schisis and lamellar schisis, but not peripheral schisis, FPS = foveo‐peripheral type patients who had foveal and peripheral schisis without lamellar schisis, FS = foveal schisis, MA = macular atrophy.

Collectively, 25 distinct *RS1* variants (NM_000330.3) were identified, of which eight were novel (c.577C>A, c.14 delT, c.554C>T, c.416 A>G, c.52+2T>A, c.656G>A, c.577_579del and c.325G>A) and one was *de novo* (c.626G>T; Table 1). Analysis of the potential pathogenicity of the novel variants is shown in Table S2. Variants were distributed from exon 1 to exon 6 and were of a wide range of mutation types, including twenty (66.67%) missense, six (20%) frameshift, two splicing and two copy‐number variants (Fig. 1A). All missense mutations were localized in the discoidin domain site, which is encoded by exons 6 (55%, *n* = 11), 4 (27.27%, *n* = 6) and 5 (13.64 %, *n* = 3; Fig. 1B). No patients had any pathogenic or likely pathogenic mutations associated with other inherited eye diseases.

### Database analysis of the reported variants

To date, a total of 289 pathologic *RS1* mutations have been identified in the HGMD, distributed from exon 1 to exon 6 (Fig. S1). Among them, 51.90% are missense, 17.30% are frameshift, 10.38% are nonsense, 11.42% are gross deletions or insertions, and 8.65 % are splicing effect (Fig. S1A). Of all the identified variants with small base mutations (*n* = 255, missense, frameshift, nonsense, splicing), 77.65% (*n* = 198) are localized in the discoidin domain site (Fig. S1B), which is encoded by exons 5 (29.41%, *n* = 75), 6 (26.27%, *n* = 67) and 4 (23.14%, *n* = 59).

### Revision and refinement of initial clinical diagnosis and risk to family members

After genetic analysis, 30 patients received a definite diagnosis of XLRS. We successfully revised the clinical diagnosis of three patients (10%), who were initially diagnosed with macular hole, cystoid macular oedema and macular dystrophy. Of all the patients in this study, 76.67% (*n* = 23) had no known family history, and 23.33% (*n* = 7) had at least one other affected relative: four were presumed to be inherited in an autosomal recessive manner, while three were presumed to show X‐linked recessive inheritance. However, the revised distribution of inheritance modes showed that all 30 patients had X‐linked recessive inheritance, of whom 29 had inherited the mutation from their mothers and one carried a *de novo* mutation.

### Clinical findings

Table 1 summarizes the clinical characteristics of all patients in the present study. The mean BCVA was 0.28 ± 0.17 (*n* = 60; range, 20/1000–20/25), and 51.67% (31/60) of eyes had a BCVA better than 20/67, while 88.33% (53/60) of eyes had a BCVA better than 20/200. The mean BCVA with patients younger than 20 years (*n* = 21; mean age, 8.81 ± 4.14 years) was 0.33 ± 0.17 (range, 0.03–0.8; median, 0.3), and the mean BCVA with patients older than 20 years (*n* = 9; mean age, 31.89 ± 8.42 years) was 0.17 ± 0.10 (range, 0.02–0.3; median, 0.12), which is worse than BCVA with patients younger than 20 years (p < 0.001). Refractive error was found in all eyes, with 51.67% (*n* = 31 eyes) having hyperopia (+2.84 ± +1.80; range, +0.55D to +9.25D), and 36.67% (*n* = 22 eyes) having myopia (−1.51 D ± −0.67 D; range, −0.5 D to −5.0 D).

### Tomographic structural changes

**Fig. 2:**
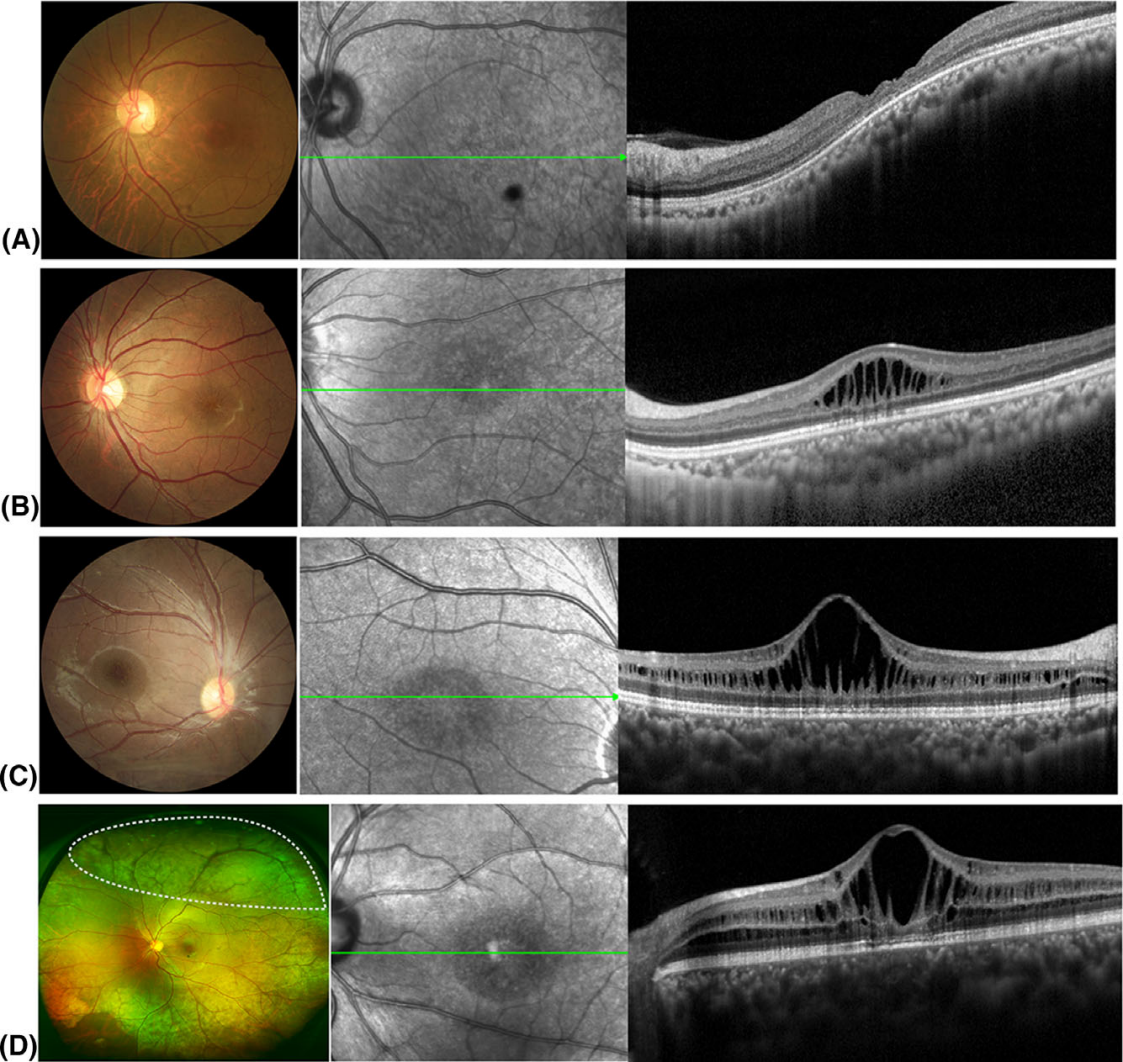
Representative fundus photographs and optical coherence tomography (OCT) images depicting different types of retinoschisis. (A) Fundus photographs and OCT findings of patients with macular atrophy. (B) Fundus photographs and OCT findings of foveal schisis (FS) patients with foveal schisis only. (C) Fundus photographs and OCT findings of foveo‐lamellar (FLS) patients with foveal schisis and lamellar schisis but no peripheral schisis. (D) Fundus photographs and OCT findings of complex type (FLPS) patients with foveal, lamellar and peripheral schisis. The white dotted area on the ultra‐wide‐field fundus photographs shows the presence of peripheral schisis.

In this study, SD‐OCT data were available for all 60 eyes. Four eyes (6.67%, 4/60) showed macular atrophy (MA) without schisis (Fig. 2A), while all others had FS (93.33%, 56/60; Fig. 1C). Subjects with MA were older than those with FS (42.5 ± 6.81 versus 12.96 ± 8.98 years, respectively). Of all eyes presenting with FS, 52 (92.86%) also had LS and 35 (62.5%) had PS. In detail, 33 eyes (58.93%; mean age, 13.78 ± 9.09 years) had FLPS (Fig. 2D), 19 eyes (33.93%; mean age, 16.79 ± 12.57 years) had FLS (Fig. 2C), two eyes (3.57%; age 7) had FPS, and two eyes (3.57%; age 24) had FS (Fig. 2B). There was no significant correlation between schisis type and patient age (p > 0.05). The average central macular thickness was 492.37 ± 150.45 µm (range, 143–1031 µm). Schisis cavities were mainly localized in the INL (56/56, 100.00%), ONL (46/56, 82.14%), GCL (35/56, 62.50%) and outer plexiform layer (OPL; 31/56, 55.36%; Table 2 and Table S3). Schisis changes involved four layers in 22 eyes (39.29%; mean age, 15.17 ± 9.50 years), three layers in 14 eyes (25%; mean age, 10.17 ± 5.78 years), two layers in 18 eyes (32.14%; mean age, 12.75 ± 10.46 years), and one layer in two eyes of one patient (5.36%; age 5 years). There was no significant correlation between layer involvement and age of these patients (Table 1).

**Table 2:** Complication characteristics of studied eyes in this cohort of patients.

Complications	Data	XLRS type	Average age ± SD (range) (years)
Vascular abnormality	27 (45%)	FLPS (24), MA (3)	14.59 ± 12.06 (5–48)
Vitreous veils	24 (40%)	FLPS (24)	13.50 ± 8.38 (5–34)
Pigment changes	24 (40%)	FLPS (21), MA (3)	15.13 ± 12.42 (4–48)
Exudations	14 (23.33%)	FLPS (12), MA (2)	16.25 ± 14.15 (5–48)
Inner retinal break	6 (10%)	FLPS (6)	16.5 ± 8.07 (9–27)
Glaucoma	6 (10%)	FLPS (3), FLS (3).	23 ± 10.84 (10–34)
Macular atrophy	4 (6.67%)	MA (4)	42.5 ± 6.81 (34–48)
Cataract	4 (6.67%)	FLPS (4)	17 ± 9.24 (9–25)
Retinal detachment	2 (3.33%)	FLPS (2)	9 (L), 25 (L)
Inner macular hole	2 (3.33%)	FLS (2)	10 (L), 40 (L)
Vitreous haemorrhage	2 (3.33%)	FLPS/RD (2)	9 (L), 5 (R)
Strabismus	2 (3.33%)	FPS (2)	7 (OU)
Morning glory	2 (3.33%)	FLS (2)	33 (OU)
Retinal white flecks	1 (1.67%)	FLPS (1)	9 (L)

FLPS = foveo‐lamellar schisis, plus peripheral schisis, FLS = foveo‐lamellar schisis, FS = foveal schisis, L = left, MA = macular atrophy, OU = binocular, R = right, RD = retinal detachmentSD = standard deviation, XLRS = X‐linked retinoschisis.

### Electrophysiology

Full‐field electroretinography (ffERG) results were available for 14 eyes of seven patients (Table S4). Electroretinography (ERG) demonstrated reduced b‐wave amplitudes in all eyes (100%), reduced a‐wave amplitudes in three eyes (10.71%), and reduced *a*‐ and *b*‐wave amplitudes in three eyes (10.71%). The *b*/*a* ratio was reduced (<1.0) in 12 eyes (85.71%).

### Ocular comorbidities

**Fig. 3:**
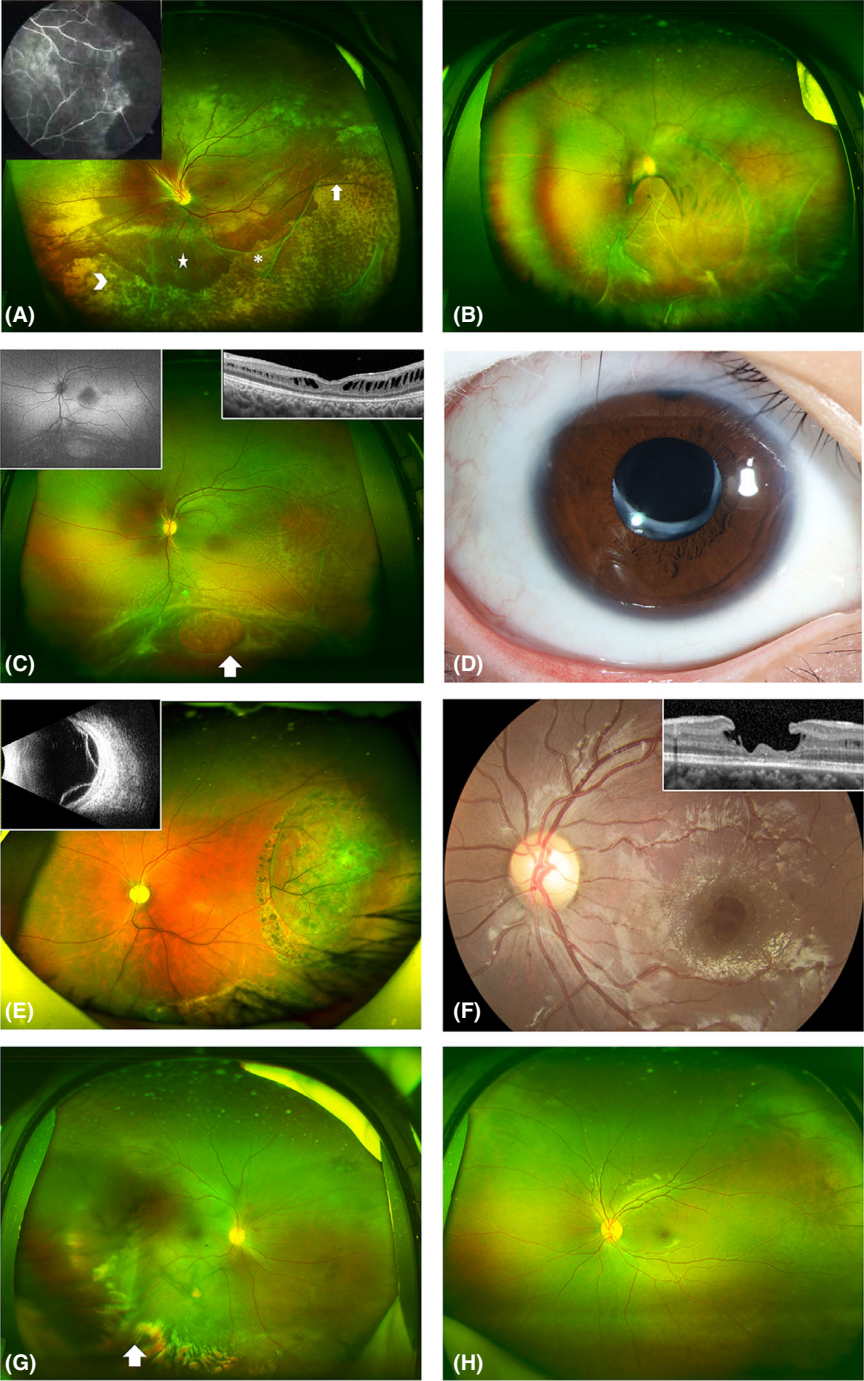
Representative ultra‐wide‐field fundus appearance depicting complications in patients with X‐linked retinoschisis. (A) Patient with peripheral schisis and multiple vitreous veils (asterisk) connected to the retina with bridging vessels (white arrows). A large amount of yellow‐white exudation (arrowhead) is seen at the inferior temporal area. The five‐pointed star at the inferior area indicates vascular sheathing. Peripheral retinal schisis at the superior area causes distortion of the optic disc. The image in the upper left corner is the corresponding fluorescein angiogram, which shows large areas of nonperfusion at the peripheral retina. (B) Patient with retinal detachment at the inferior temporal area, and multiple vitreous veils connected to the retina with bridging and tortuous vessels. A pigmented demarcation line is seen at the posterior pole. An occluded blood vessel appears as a white line at the inferior area. (C) Patient with typical white spots in the posterior pole, and multiple vitreous veils connected to the retina with bridging vessels. White arrow indicates a large inner retinal break. The image in the upper left corner is the corresponding autofluorescence, which shows hypoautofluorescence at the fovea area. The image in the upper right corner shows corresponding OCT changes. OCT shows lamellar schisis and mild macular atrophy. (D) Patient with cataract after intraocular lens implantation. (E) Patient with bullous retinal detachment after retinal photocoagulation. The image in the upper left corner is the corresponding ultrasound B image. (F) Representative ultra‐wide‐field fundus of a patient with an inner macular hole. The image in the upper right corner shows corresponding OCT changes. OCT shows an inner macular hole with an intact outer structure of the retina. (G) Patient with mild vitreous haemorrhage. (H) Patient with a typical cartwheel‐like appearance in the central macula and peripheral retinal splitting at the inferior area.

Complications seen in this cohort of patients are shown in Tables 1 and 2 and Fig. 1D. Vascular abnormalities (VAAs; Fig. 3A,B) occur most frequently (*n* = 27, 45%; mean age, 14.59 ± 12.06 years), including vascular sheathing, retinal nonperfusion, vascular white line and haemorrhagic spots caused by vessel twisting. Most of the eyes with VAAs had FLPS (88.89%, *n* = 24), and the others had MA (11.11%, *n* = 3). Vitreous veils (VVs; Fig. 3A–C) were observed in 24 of 60 eyes (40%), all of which were FLPS (mean age, 13.50 ± 8.38 years). Pigment changes (Fig. 3B) were seen in 24 eyes (40%; mean age, 15.13 ± 12.42 years), of which 21 were FLPS and three were MA. Exudation (Fig. 3A) was found in 14 eyes (23.33%; mean age, 16.25 ± 14.15 years), of which twelve were FLPS and two were MA. Inner retinal breaks (Fig. 3C) were found in six eyes (10%; mean age, 16.5 ± 8.07), all of which were FLPS. Glaucoma was found in six eyes of three patients (10%), of whom two (FLPS 1, age 34 years; FLS 2, age 10 years) were primary open‐angle glaucoma (POAG), and one (FLPS, age 25 years) was closed‐angle glaucoma. Cataracts (Fig. 3D) were found in four eyes (6.67%) of three patients (FLPS); one patient (age 25 years) also had PCAG, one (age 9 years) had RD, and one (age 9 years) had RD, pigment changes, exudation and VAAs. Retinal detachment (RD; Fig. 3E) occurred in two eyes (3.33%) from two patients, one aged 9 and one aged 25, both of whom were FLPS. Inner MH (IMH; Fig. 3F) was identified in two eyes (FLPS) from two patients (age 10 and 40 years). Vitreous haemorrhage (VH; Fig. 3G) was identified in two eyes (FLPS) from two patients, both of whom also had RD. Retinal white flecks (Fig. 3C) were found in one eye (FLPS) of a 9‐year‐old boy who also had VAAs, RD and VVs. In total, 94.29% (*n* = 33) of eyes with PS (*n* = 35) showed different degrees and types of complications, while the other two patients were only aged 4 and 6 years. However, of the 21 eyes without PS, only two eyes (9.52%) from two patients had complications (IMH). No significant difference was found between the ages of the patients and the degrees or types of complications (Table 1).

### Genotype–phenotype correlations

To determine whether these clinical findings were associated with specific mutations, or types or locations of mutations, we further analysed the relationship between structural changes and ocular complications with specific mutations (Table 1). We found that the clinical findings did not correlate with specific *RS1* variant or mutation types. Different individuals, even with the same mutation, were found to show completely different structural changes and complications (e.g. F2 and F8, F3 and F4, F12 and F19, and F16 and F27).

## Discussion

We herein provide detailed and comprehensive reports of the genetic and clinical characteristics of a cohort of 30 unselected Chinese male patients with XLRS. We identified 25 distinct *RS1* variants, of which eight were novel. The large proportion of novel mutations and variable types of causative variants suggest that the *RS1* mutational spectrum in the Chinese population is distinct from that in other ethnic groups. Perhaps there may be many *RS1* mutations remain to be identified and confirmed in different races, which will be vital for pathogenicity judgement and genetic counselling.

A *de novo* mutation in *RS1* was first identified by Gehrig et al. (1999a) and Gehrig et al. (1999b) (c.608C>T Pro203Leu), and then, another two *de novo* mutations were reported in 2009: c.238C>T p.Gln80Ter and c.2I4G>A p.Glu72Lys (Riveiro‐Alvarez et al. 2009). It has been suggested that *de novo* mutations mainly occur in CG dinucleotides (Riveiro‐Alvarez et al. 2009). Consistent with this, we identified the fourth *de novo* mutation in *RS1*, c.626G>T p.Arg209Leu, which is also located within CG dinucleotides, and representing a frequency of 3.33% (1/30). These results indicate that *de novo* mutations play a vital role in XLRS, and attention should be paid to them because they may have important implications in obtaining a correct genetic diagnosis and in enabling more accurate genetic counselling.

By analysing the reported variants and those identified in the present study, we found that most mutations were localized in the discoidin domain and that missense mutations were the most common type (Hu et al. 2017; Chen et al. 2020). Of all reported variants, 11.76% are gross deletions or insertions, which may not be detectable by NGS. This has important guidance for the selection of sequencing methods used to diagnose XLRS, especially when small mutation analyses show negative results.

The mean BCVA of patients younger than 20 years was 0.33 ± 0.17, and it is better than that of patients older than 20 years. These data indicate that the optimal intervention window for a possible therapeutic approach is within the first 2 decades of life. We report PS in 58.33% (35/60) of eyes in our cohort, which is similar to previously reported (ranged from 43% to 79%) (Prenner et al. 2006; Wang et al. 2015; Chen et al. 2020). The rate was generally higher than the rate (48%) reported recently by Chen et al. (2020), who studied a large series of Chinese patients affected by XLRS. One possible reason is that ultra‐wide‐field fundus imaging was used for retinal examination in this study, which enabled a more detailed identification of the peripheral retinal changes, and for identification of unnoticed peripheral retinoschisis (Leshno et al. 2019; Cicinelli et al. 2020). The most common type of XLRS in this study was FLPS, followed by FLS, then FS and FPS, which is similar to earlier findings (Ores et al. 2018). The mechanisms that leading to the occurrence of different types of schisis are not well understood, and we propose the possible reasons are the distribution and secretion of retinoschisin protein. We identified schisis changes mainly in the INL and ONL, followed by the GCL and OPL, which is in agreement with previous studies (Ores et al. 2018). These data indicate that retinoschisin is widely distributed in the retina. However, further analysis revealed no significant correlations between schisis type, layer involvement, patient age and mutations, suggesting the existence of more complex regulatory mechanisms responsible for schisis variations.

X‐linked juvenile retinoschisis (XLRS) patients have a high incidence of various complications, with VAAs, VVs and pigment changes the most common in the present study, and the incidence is higher than reported in Caucasians (Pennesi et al. 2018). This may be attributed to the higher rate of PS in our study (58.33% versus 25%). Optical coherence tomography (OCT) angiography‐based assessment previously found that microvascular changes were mainly located in the deep capillary plexus and that enlargement of the foveal avascular zone area correlated with BCVA decline (Romano et al. 2019). However, it remains unclear whether this is the cause (originating directly from a mutation) or the outcome (caused by other lesions such as cystic impairment). Vitreous veils (VVs) are common in XLRS and result from the occurrence of larger atrophic inner breaks in the thin inner schisis cavities (Campbell et al. 2015), which are often associated with VH, RD and VAAs (Ambler & Gutman 1991). Exudation and IRB are not rare in XLRS and may occur from the wrinkling or traction of macular detachment, abnormal vascular proliferation, or traction of VVs (Hu et al. 2017; Wood et al. 2019). Neovascular glaucoma has also previously been reported in XLRS (Ando et al. 2000), and with some studies attributing it to RD or extensive schisis (Manschot 1972), while others report that ischaemia in ocular tissues is the main cause (Ando et al. 2000). Although we did not observe neovascular glaucoma in our study, we detected two cases with POAG and one with PCAG; current information is insufficient to determine whether they are complication or simply coexist with XLRS. Macular atrophy (MA) was found in four eyes of three patients who were older than other patients; it was associated with a thinner ONL, which is consistent with previous results (Ores et al. 2018). Retinal detachment (RD) was only found in two eyes from two patients, one aged 9 and the other aged 25; the rate reported here is lower than the 5.5%–16% documented in previous reports (Fahim et al. 2017). Both IMH and VH were identified in two eyes from two patients in our study. The occurrence of VH at a young age is consistent with the report of its development before the age of 10 (Kellner et al. 1990). However, IMH can occur at any stage. Retinal white flecks in the posterior pole were seen in one eye in our study, and have also been reported in previous studies (Fahim et al. 2017). No second pathogenic variants associated with inherited eye diseases, including those in *RDH5*, *RHO*, *RLBP1* and PRPH2, were found in the affected patient. This unusual fundus finding could be a rare characteristic of XLRS, though larger samples are needed for further verification. The prevailing viewpoint is that PS increases the likelihood of these complications occurring (Fahim et al. 2017; Wood et al. 2019). Our data showed that 94.29% of eyes with PS had different degrees and types of complications, while only 9.52% of eyes without PS had other abnormalities. However, future complications should not be ruled out in these patients with PS because they are still young (aged 4 and 6 years). Therefore, close follow‐up is needed. It should also be noted that morning glory disc anomaly and POAG occurred as abnormalities in five eyes, although they may simply coexist with FLS.

Limitations of this study include the small size of our cohort, and the lack of a longitudinal follow‐up to investigate morphofunctional correlations and changes. However, because XLRS is relatively rare, it is difficult to collect a large study group, and multimodal images are hard to acquire, especially from young patients.

## Conclusions

This study provides comprehensive analyses of the genetic and clinical characteristics of XLRS in a cohort of Chinese patients. We show that the most prevalent mutation type is missense mutation, and the hot spot is localized in the discoidin domain. The fourth de novo mutation in *RS1* was identified. X‐linked juvenile retinoschisis (XLRS) has a wide spectrum of clinical characteristics; hence, molecular diagnosis is crucial for its diagnosis and genetic counselling. Peripheral schisis (PS) is a risk factor for the high incidence of complications, and closer follow‐up may be beneficial. And no clear genotype–phenotype correlations were found. Our results not only have guiding value for selecting sequencing methods used for XLRS patients and genetic counselling, but also have an important clinical impact that should be considered in the perspective of gene therapy for XLRS patients.

## Supporting information

**Fig. S1.** Overview of pathologic *RS1* mutations reported in the Human Gene Mutation Database. (A) Number of *RS1* mutations of different types reported in the Human Gene Mutation Database. (B) Distribution of *RS1* variants reported in the Human Gene Mutation Database. Exons are numbered in black font, and introns in blue font. Total mutations are shown in red font.

**Table S1.** Details of the 762 genes in the panel.

**Table S2.** Analysis of the potential pathogenicity of the novel variants.

**Table S3.** Schisis localization of studied eyes in this cohort of patients.

**Table S4.** Electrophysiological characteristics of X‐linked juvenile retinoschisis patients.

## Data Availability

All data relevant to the study are included in the article or uploaded as supplementary information.

## References

[aos14642-bib-0001] AA (1998): Functional implications of the spectrum of mutations found in 234 cases with X‐linked juvenile retinoschisis. The Retinoschisis Consortium. Human Mol Genet 7: 1185–1192. 9618178 10.1093/hmg/7.7.1185

[aos14642-bib-0002] Ambler JS & Gutman FA (1991): Retinal detachment and retinoschisis. Ophthalmology 98: 1. 10.1016/s0161-6420(91)32353-4 2023722

[aos14642-bib-0003] Ando A, Takahashi K, Sho K, Matsushima M, Okamura A & Uyama M (2000): Histopathological findings of X‐linked retinoschisis with neovascular glaucoma. Graefes Arch Clin Exp 238: 1–7. 10.1007/s004170050001 10664045

[aos14642-bib-0004] Bowles K, Cukras C, Turriff A, Sergeev Y, Vitale S, Bush RA & Sieving PA (2011): X‐linked retinoschisis: RS1 mutation severity and age affect the ERG phenotype in a cohort of 68 affected male subjects. Invest Ophthalmol Vis Sci 52: 9250–9256. 22039241 10.1167/iovs.11-8115 PMC3302432

[aos14642-bib-0005] Campbell JP, Skalet AH & Lauer AK (2015): Vitreous veils associated with congenital X‐linked retinoschisis. JAMA Ophthalmol 133: e151155. 26270406 10.1001/jamaophthalmol.2015.1155

[aos14642-bib-0006] Chen C, Xie Y, Sun T, Tian L, Xu K, Zhang X & Li Y (2020): Clinical findings and RS1 genotype in 90 Chinese families with X‐linked retinoschisis. Mol Vis 26: 291–298. 32300273 PMC7155891

[aos14642-bib-0007] Chen J, Xu K, Zhang X, Pan Z, Dong B & Li Y (2014): Novel mutations of the RS1 gene in a cohort of Chinese families with X‐linked retinoschisis. Mol Vis 20: 132–139. 24505212 PMC3913487

[aos14642-bib-0008] Chu Y, Fang D, Hou QF, Wang LY, Guo XR, Wang YT & Liao SX (2013): [Detection and prenatal diagnosis for RS1 gene mutations in two Chinese families with X‐linked juvenile retinoschisis]. Zhonghua Yi Xue Yi Chuan Xue Za Zhi 30: 199–202. 23568735 10.3760/cma.j.issn.1003-9406.2013.04.017

[aos14642-bib-0009] Cicinelli MV, Marchese A, Bordato A, Manitto MP, Bandello F & Battaglia Parodi M (2020): Reviewing the role of ultra‐widefield imaging in inherited retinal dystrophies. Ophthalmol Ther 9: 249–263. 32141037 10.1007/s40123-020-00241-1 PMC7196101

[aos14642-bib-0010] Fahim AT, Ali N, Blachley T & Michaelides M (2017): Peripheral fundus findings in X‐linked retinoschisis. Br J Ophthalmol 101: 1555–1559. 28348004 10.1136/bjophthalmol-2016-310110

[aos14642-bib-0011] Gao FJ, Li JK, Chen H et al. (2019a): Genetic and clinical findings in a large cohort of Chinese patients with suspected retinitis pigmentosa. Ophthalmology 126: 1549–1556. 31054281 10.1016/j.ophtha.2019.04.038

[aos14642-bib-0012] Gao FJ, Qi YH, Hu FY et al. (2019b): Mutation spectrum of the bestrophin‐1 gene in a large Chinese cohort with bestrophinopathy. Br J Ophthalmol 104: 846–851. 31519547 10.1136/bjophthalmol-2019-314679

[aos14642-bib-0013] Gehrig A, Weber BH, Lorenz B & Andrassi M (1999a): First molecular evidence for a de novo mutation in RS1 (XLRS1) associated with X linked juvenile retinoschisis. J Med Genet 36: 932–934. 10636740 PMC1734280

[aos14642-bib-0014] Gehrig A, White K, Lorenz B, Andrassi M, Clemens S & Weber BH (1999b): Assessment of RS1 in X‐linked juvenile retinoschisis and sporadic senile retinoschisis. Clin Genet 55: 461–465. 10450864 10.1034/j.1399-0004.1999.550611.x

[aos14642-bib-0015] Hewitt AW, FitzGerald LM, Scotter LW, Mulhall LE, McKay JD & Mackey DA (2005): Genotypic and phenotypic spectrum of X‐linked retinoschisis in Australia. Clin Exp Ophthalmol 33: 233–239. 15932525 10.1111/j.1442-9071.2005.01018.x

[aos14642-bib-0016] Hotta Y, Fujiki K, Hayakawa M et al. (1998): Japanese juvenile retinoschisis is caused by mutations of the XLRS1 gene. Hum Genet 103: 142–144. 9760195 10.1007/pl00008705

[aos14642-bib-0017] Hu QR, Huang LZ, Chen XL, Xia HK, Li TQ & Li XX (2017): Genetic analysis and clinical features of X‐linked retinoschisis in Chinese patients. Sci Rep 7: 44060. 28272453 10.1038/srep44060 PMC5341047

[aos14642-bib-0018] Huang XY, Zhuang H, Wu JH et al. (2017): Targeted next‐generation sequencing analysis identifies novel mutations in families with severe familial exudative vitreoretinopathy. Mol Vis 23: 605–613. 28867931 PMC5568910

[aos14642-bib-0019] Huopaniemi L, Fellman J, Rantala A, Eriksson A, Forsius H, De La Chapelle A & Alitalo T (1999): Skewed secondary sex ratio in the offspring of carriers of the 214G > A mutation of the RS1 gene. Ann Hum Genet 63: 521–533. 11246454 10.1017/S0003480099007812

[aos14642-bib-0020] Ip M, Garza‐Karren C, Duker JS, Reichel E, Swartz JC, Amirikia A & Puliafito CA (1999): Differentiation of degenerative retinoschisis from retinal detachment using optical coherence tomography. Ophthalmology 106: 600–605. 10080221 10.1016/S0161-6420(99)90123-9

[aos14642-bib-0021] Kellner U, Brummer S, Foerster MH & Wessing A (1990): X‐linked congenital retinoschisis. Graefes Arch Clin Exp Ophthalmol 228: 432–437. 2227486 10.1007/BF00927256

[aos14642-bib-0022] Leshno A, Barak A, Barzelay A, Zloto O & Neudorfer M (2019): Diagnosis of peripheral retinoschisis using ultrasound biomicroscopy. Ophthalmic Surg Lasers Imaging Retina 50: e196–e202. 31415704 10.3928/23258160-20190806-12

[aos14642-bib-0023] Li X, Ma X & Tao Y (2007): Clinical features of X linked juvenile retinoschisis in Chinese families associated with novel mutations in the RS1 gene. Mol Vis 13: 804–812. 17615541 PMC2768756

[aos14642-bib-0024] Manschot WA (1972): Pathology of hereditary juvenile retinoschisis. Arch Ophthalmol 88: 131–138. 5054298 10.1001/archopht.1972.01000030133002

[aos14642-bib-0025] Molday LL, Hicks D, Sauer CG, Weber BH & Molday RS (2001): Expression of X‐linked retinoschisis protein RS1 in photoreceptor and bipolar cells. Invest Ophthalmol Vis Sci 42: 816–825. 11222545

[aos14642-bib-0026] Molday RS, Kellner U & Weber BH (2012): X‐linked juvenile retinoschisis: clinical diagnosis, genetic analysis, and molecular mechanisms. Prog Retin Eye Res 31: 195–212. 22245536 10.1016/j.preteyeres.2011.12.002 PMC3334421

[aos14642-bib-0027] Ores R, Mohand‐Said S, Dhaenens CM et al. (2018): Phenotypic characteristics of a french cohort of patients with X‐linked retinoschisis. Ophthalmology 125: 1587–1596. 29739629 10.1016/j.ophtha.2018.03.057

[aos14642-bib-0028] Pennesi ME, Birch DG, Jayasundera KT et al. (2018): Prospective evaluation of patients with X‐linked retinoschisis during 18 months. Invest Ophthalmol Vis Sci 59: 5941–5956. 30551202 10.1167/iovs.18-24565 PMC6295939

[aos14642-bib-0029] Pimenides D, George ND, Yates JR, Bradshaw K, Roberts SA, Moore AT & Trump D (2005): X‐linked retinoschisis: clinical phenotype and RS1 genotype in 86 UK patients. J Med Genet 42: e35. 15937075 10.1136/jmg.2004.029769 PMC1736077

[aos14642-bib-0030] Prenner JL, Capone A Jr, Ciaccia S, Takada Y, Sieving PA & Trese MT (2006): Congenital X‐linked retinoschisis classification system. Retina 26: S61–S64. 16946682 10.1097/01.iae.0000244290.09499.c1

[aos14642-bib-0031] Renner AB, Kellner U, Fiebig B, Cropp E, Foerster MH & Weber BH (2008): ERG variability in X‐linked congenital retinoschisis patients with mutations in the RS1 gene and the diagnostic importance of fundus autofluorescence and OCT. Doc Ophthalmol 116: 97–109. 17987333 10.1007/s10633-007-9094-5

[aos14642-bib-0032] Riveiro‐Alvarez R, Trujillo‐Tiebas MJ, Gimenez A, Cantalapiedra D, Vallespin E, Aguirre‐Lamban J, Villaverde C & Ayuso C (2007): Novel human pathological mutations. Gene symbol: RS1. Disease: X‐linked juvenile retinoschisis. Human Genet 121: 647. 17879437

[aos14642-bib-0033] Riveiro‐Alvarez R, Trujillo‐Tiebas MJ, Gimenez‐Pardo A et al. (2009): Correlation of genetic and clinical findings in Spanish patients with X‐linked juvenile retinoschisis. Invest Ophthalmol Vis Sci 50: 4342–4350. 19324861 10.1167/iovs.09-3418

[aos14642-bib-0034] Romano F, Arrigo A, Ch'ng SW, Battaglia Parodi M, Manitto MP, Martina E, Bandello F & Stanga PE (2019): Capillary network alterations in x‐linked retinoschisis imaged on optical coherence tomography angiography. Retina 39: 1761–1767. 29877903 10.1097/IAE.0000000000002222

[aos14642-bib-0035] Sauer CG, Gehrig A, Warneke‐Wittstock R et al. (1997): Positional cloning of the gene associated with X‐linked juvenile retinoschisis. Nat Genet 17: 164–170. 9326935 10.1038/ng1097-164

[aos14642-bib-0036] Shinohara K, Tanaka N, Jonas JB, Shimada N, Moriyama M, Yoshida T & Ohno‐Matsui K (2018): Ultrawide‐field OCT to investigate relationships between myopic macular retinoschisis and posterior staphyloma. Ophthalmology 125: 1575–1586. 29716783 10.1016/j.ophtha.2018.03.053

[aos14642-bib-0037] Skorczyk A & Krawczynski MR (2012): Four novel RS1 gene mutations in Polish patients with X‐linked juvenile retinoschisis. Mol Vis 18: 3004–3012. 23288992 PMC3534142

[aos14642-bib-0038] Splinter K, Adams DR, Bacino CA et al. (2018): Effect of genetic diagnosis on patients with previously undiagnosed disease. N Engl J Med 379: 2131–2139. 30304647 10.1056/NEJMoa1714458 PMC6481166

[aos14642-bib-0039] Stenson PD, Ball EV, Mort M, Phillips AD, Shaw K & Cooper DN (2012): The Human Gene Mutation Database (HGMD) and its exploitation in the fields of personalized genomics and molecular evolution. Curr Protoc Bioinformatics Chapter 1: Unit1.13. 10.1002/0471250953.bi0113s39 22948725

[aos14642-bib-0040] Wang NK, Liu L, Chen HM et al. (2015): Clinical presentations of X‐linked retinoschisis in Taiwanese patients confirmed with genetic sequencing. Mol Vis 21: 487–501. 25999676 PMC4415592

[aos14642-bib-0041] Wood EH, Lertjirachai I, Ghiam BK et al. (2019): The natural history of congenital X‐linked retinoschisis and conversion between phenotypes over time. Ophthalmol Retina 3: 77–82. 30935660 10.1016/j.oret.2018.08.006

[aos14642-bib-0042] Yu J, Ni Y, Keane PA, Jiang C, Wang W & Xu G (2010): Foveomacular schisis in juvenile X‐linked retinoschisis: an optical coherence tomography study. Am J Ophthalmol 149: 973–978.e972. 20430364 10.1016/j.ajo.2010.01.031

