# Supplementary figures and images for: Comprehensive analysis of genetic and clinical characteristics of 30 patients with X‐linked juvenile retinoschisis in China
Source: Acta Ophthalmol. 2020 Oct 30;99(4):e470–9. doi: 10.1111/aos.14642 (PMC8359357; doi:10.1111/aos.14642)

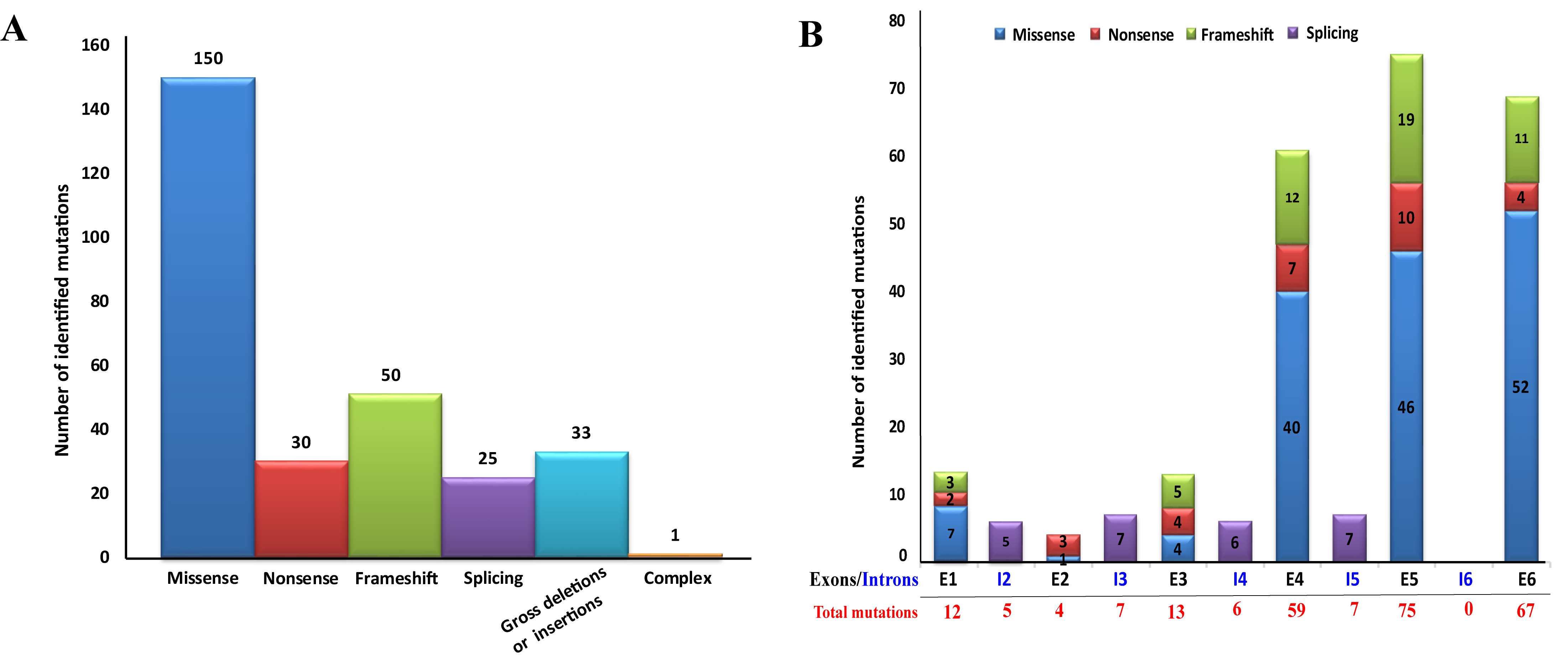

Supplement: Supplementary file 1 — Fig. S1. Overview of pathologic RS1 mutations reported in the Human Gene Mutation Database. (A) Number of RS1 mutations of different types reported in the Human Gene Mutation Database. (B) Distribution of RS1 variants reported in the Human Gene Mutation Database. Exons are numbered in black font, and introns in blue font. Total mutations are shown in red font. [file AOS-99-e470-s003.tif]
