# Supplementary material for: Comprehensive analysis of genetic and clinical characteristics of 30 patients with X‐linked juvenile retinoschisis in China
Source: Acta Ophthalmol. 2020 Oct 30;99(4):e470–9. doi: 10.1111/aos.14642 (PMC8359357; doi:10.1111/aos.14642)
Supplement: Supplementary file 3 — Table S2. Analysis of the potential pathogenicity of the novel variants. [file AOS-99-e470-s004.docx]

Supplementary table 2. Analysis of the potential pathogenicity of the novel variants.

| Nucleotide Change | SIF | LRT | PolyPhen | MutationTaster | FATHMM |
| --- | --- | --- | --- | --- | --- |
| c.577C>A p.Pro193Thr | Damaging | Deleterious | probably_damaging | Disease causing | Damaging |
| c.554C>T p.Thr185Met | Damaging | Deleterious | probably_damaging | Disease causing | Damaging |
| c.325G>A p.Gly109Arg | Tolerated | Deleterious | probably_damaging | Disease causing | Damaging |
| c.416A>G p.Gln139Arg | Damaging | Deleterious | probably_damaging | disease_causing | Damaging |
| c.656G>A p.Cys219Tyr | Damaging | Deleterious | probably_damaging | disease_causing | Damaging |
| c.52+2T>A | NA | NA | NA | disease_causing | NA |
| c.14 delT p.Ile5LysfsX121 | NA | NA | NA | NA | NA |
| c.577_579del p.Ter193del | NA | NA | NA | NA | NA |
