# Supplementary material for: Comprehensive analysis of genetic and clinical characteristics of 30 patients with X‐linked juvenile retinoschisis in China
Source: Acta Ophthalmol. 2020 Oct 30;99(4):e470–9. doi: 10.1111/aos.14642 (PMC8359357; doi:10.1111/aos.14642)
Supplement: Supplementary file 4 — Table S3. Schisis localization of studied eyes in this cohort of patients. [file AOS-99-e470-s001.docx]

Supplementary Table 3. Schisis localization of studied eyes in this cohort of patients.

| Schisis localization | Eyes (%) | XLRS Type | Average age ± SD (range) (years) | | |
| --- | --- | --- | --- | --- | --- |
| Macular schisis | 56 (93.33%) | FLPS (33), FLS (19),  FS (2), FPS (2). | 13.82±9.84 (4-40) |  |  |
| Peripheral schisis | 35 (58.33%) | FLPS (33), FPS (2) | 11.63±7.54 (4-34) |  |  |
| - GCL | 35 (62.50%) | FLPS (21), FLS (12),  FS (2) | 13.78±8.36 (5-34) |  |  |
| - INL | 56 (100.00%) | FLPS (33), FLS (19),  FS (4). | 12.96±8.98 (4-34) |  |  |
| -ONL | 46 (82.14%) | FLPS (26), FLS (16),  FS (4) | 13.57±9.81 (4-34) |  |  |
| -OPL | 31 (55.36%) | FLPS (18), FLS (9),  FS (4) | 13.94±8.66 (4-34) |  |  |
| Involving four layers | 22 (39.29%) | FLPS (13), FLS (7),  FS (2) | 15.17±9.50 (5-34) |  |  |
| -INL/ONL/OPL/GCL | 22 (39.29%） | - | - |  |  |
| Involving four layers | 14 (25.00%) | FLPS (8), FLS (4),  FS (2) | 10.17±5.78 (4-19) |  |  |
| -INL/ONL/ OPL | 6 (10.71%) | - | - |  |  |
| -INL/ONL/ GCL | 5 (8.93%) | - | - |  |  |
| -INL/OPL/GCL | 3 (5.36%) | - | - |  |  |
| Involving two layers | 18 (32.14%) | FLPS (10), FLS (8) | 12.75±10.46 (4-33) |  |  |
| -INL/ONL | 14 (25.00%） | - | - |  |  |
| -INL/GCL | 4 （7.14%） | - | - |  |  |
| Involving one layers | 2 （3.57%） | FLPS (2) | 5 |  |  |
| -INL | 2 （3.57%） | - | - |  |  |

Abbreviations. XLRS: X-linked juvenile retinoschisis. SD: standard deviation. GCL: ganglion cell layer; RNFL: retinal nerve fiber layer; IPL: inner plexiform layer; INL: inner nuclear layer; OPL: outer plexiform layer; ONL: outer nuclear layer; R: right. L: left. OU: binocular. FS: foveal schisis. FLS: foveo-lamellar schisis. FLPS: foveo-lamellar schisis, plus peripheral schisis.
