# Supplementary material for: Comprehensive analysis of genetic and clinical characteristics of 30 patients with X‐linked juvenile retinoschisis in China
Source: Acta Ophthalmol. 2020 Oct 30;99(4):e470–9. doi: 10.1111/aos.14642 (PMC8359357; doi:10.1111/aos.14642)
Supplement: Supplementary file 5 — Table S4. Electrophysiological characteristics of X‐linked juvenile retinoschisis patients. [file AOS-99-e470-s005.docx]

Supplementary Table 4. Electrophysiological Characteristics of Patients.

| Patient | Mixed Scotopic ERG a-wave Amplitude (µV) | Mixed Scotopic ERG a-wave Implicit time (msec) | Mixed Scotopic ERG b-wave Amplitude (µV) | Mixed Scotopic ERG b-wave Implicit time (msec) | Mixed Scotopic b/a wave ratio |
| --- | --- | --- | --- | --- | --- |
| F3 | 136.4/112.1 | 20/23 | 59.9 /84.7 | 50.5/50 | 0.439/0.756 |
| F5 | 90.9/101.7 | 20/18 | 43.4/47.1 | 40/38 | 0.477/0.463 |
| F6 | 142/147.4 | 18/18 | -5.3/43.9 | 52/55.5 | -0.037/0.298 |
| F7 | 94.2/118 | 18/19.5 | 126.6/161.8 | 40/42 | 1.344/1.278 |
| F8 | 88.2/127.9 | 18/17.5 | 82.4/51.4 | 46/36 | 0.934/0.402 |
| F18 | 102.2/125.6 | 19/21 | 56/68.6 | 42/45 | 0.548/0.546 |
| F20 | 251.2/260.3 | 18/18 | 16.9/86.6 | 48/55 | 0.067/0.333 |
| Normal value | 98.2-331 | 18-25 | 218-635.9 | 39-48.5 |  |

OD, right eye; OS, left eye; ERG, electroretinogram; μV, microvolts.
